# Bone serves as a transfer station for secondary dissemination of breast cancer

**DOI:** 10.1038/s41413-023-00260-1

**Published:** 2023-04-21

**Authors:** Yufan Huang, Hongli Wang, Xiaomin Yue, Xiaoqing Li

**Affiliations:** 1 Department of Biochemistry and Molecular Biology, Tianjin Medical University Cancer Institute and Hospital, National Clinical Research Center of Cancer, Tianjin, 300060 China; 2 Tianjin’s Clinical Research Center for Cancer, Tianjin Medical University Cancer Institute and Hospital, National Clinical Research Center of Cancer, Tianjin, 300060 China; 3 Key Laboratory of Breast Cancer Prevention and Treatment of the Ministry of Education, Tianjin Medical University Cancer Institute and Hospital, National Clinical Research Center of Cancer, Tianjin, 300060 China

**Keywords:** Cancer, Bone

## Abstract

Metastasis is responsible for the majority of deaths among breast cancer patients. Although parallel polyclonal seeding has been shown to contribute to organ-specific metastasis, in the past decade, horizontal cross-metastatic seeding (metastasis-to-metastasis spreading) has also been demonstrated as a pattern of distant metastasis to multiple sites. Bone, as the most frequent first destination of breast cancer metastasis, has been demonstrated to facilitate the secondary dissemination of breast cancer cells. In this review, we summarize the clinical and experimental evidence that bone is a transfer station for the secondary dissemination of breast cancer. We also discuss the regulatory mechanisms of the bone microenvironment in secondary seeding of breast cancer, focusing on stemness regulation, quiescence-proliferation equilibrium regulation, epigenetic reprogramming and immune escape of cancer cells. Furthermore, we highlight future research perspectives and strategies for preventing secondary dissemination from bone.

## Introduction

Distant metastasis is the leading cause of death in breast cancer patients. The metastasis of breast cancer exhibits organotropism, most frequently to bone, followed by the lungs and, less frequently, the liver and brain. Because of the heterogeneity of cancer cells, multiple distant metastases may parallelly originate from the polyclonal organ-specific seeding of primary tumor cells.^1,2^ This parallel polyclonal model is supported by genomic sequencing-based evolutionary phylogenetic analyses^2–4^ and evidence that breast cancer cells with different genetic profiles exhibit marked differences in their ability to colonize various metastatic sites.^5,6^ Moreover, horizontal cross-metastatic seeding (metastasis-to-metastasis spreading) has been demonstrated in molecular evolutionary models and phylogenetic analyses in the past decade.^2,7–11^ Characterization of the genomic evolutionary history of metastatic cancers using whole-genomic sequencing has revealed secondary seeding to other sites from axillary lymph node metastasis,^9^ ovarian metastasis^10^ and bone metastasis^9^ in breast cancer and prostate cancer patients.

Bone is the most frequent destination of metastatic breast cancer cells and is also the most common site of first distant relapse of breast cancer.^12,13^ Breast cancer cells that spread to the bone form bone metastases or reside in bone marrow as dormant disseminated tumor cells (DTCs) or micrometastases. In bone, the fates of cancer cells are determined by the interaction of cancer cells with resident cells and cytokines in the bone microenvironment. Cancer cells not only educate the bone microenvironment to become a suitable soil for their survival^14,15^ but also represent metastatic seeds for secondary dissemination invigorated by the bone microenvironment.^16^ Here, we present a review on the role of the bone microenvironment in facilitating the secondary dissemination of breast cancer, focusing on clinical and experimental evidence and the underlying mechanisms.

### Clinical evidence of bone as a transfer station for secondary dissemination

Clinical studies have suggested that bone may not be the final destination of breast cancer cells that seed in the bone. Initial bone-only dissemination of breast cancer has been indicated to correlate with a high risk of subsequent multiorgan relapse. Moreover, adjuvant therapies eliminating bone marrow tumor cells contribute to a reduction in the risk of extraosseous metastases in patients with bone marrow DTCs or bone metastasis. These clinical data support the hypothesis that bone serves as a transfer site for secondary dissemination.

#### Initial bone metastasis increases the risk of secondary dissemination

Although multiple organs are involved in the first relapse diagnosis in some patients, single-organ metastasis at the first relapse diagnosis is found in more than 70% of patients.^13,17,18^ Clinical studies have shown that bone is the most common site of first distant relapse.^12,13,19^ For patients with distant metastasis, 71.8% experienced relapse in bone, which is significantly higher than the proportion of patients with visceral relapse in the lung (35.9%), liver (20.5%) or brain (6.4%) based on a cohort of 1 459 breast cancer patients.^20^ In a clinical study of 2 240 breast cancer patients carried out by Coleman RE and colleagues, almost half of the patients with a first relapse in bone subsequently exhibited relapse in other sites (soft tissue, liver, pleura, lung and brain).^21^ The same research group further reported a 62% subsequent incidence of extraosseous metastasis following initial bone metastasis based on a cohort of 367 breast cancer patients whose first site of distant metastasis was bone.^12^ In another retrospective clinical study, 52.4% of breast cancer patients with bone-only metastasis developed secondary distant metastasis within a median metastasis‑free interval of 21 months.^22^ The most common secondary metastatic site was the liver (51.4%), followed by the lung (30.3%) and brain (13.8%).^22^ These clinical data imply the possibility of secondary dissemination from the initial bone metastasis site to other sites.

#### The presence of DTCs and micrometastasis in the bone marrow correlates with high risks of relapse in extraosseous organs

Not all breast cancer cells that spread into bone immediately develop into bone metastases. Instead, they may reside in the bone marrow as inactive DTCs or micrometastases.^23^ Bone marrow DTCs or micrometastases have been detected at diagnosis or during follow-up in 15% to 30% of breast cancer patients without other organ metastases.^24–26^ The presence of DTCs or micrometastases is a significant prognostic factor for poor overall survival (OS) and disease-free survival (DFS). In a clinical study of 3 141 breast cancer patients reported by Tuebingen University, bone marrow DTCs were confirmed as an independent predictor of OS and DFS, and patients with DTCs exhibited increased risks of relapse and death compared to DTC-negative (DTC^–^) patients.^27^ Almost 50% of bone marrow DTC-positive (DTC^+^) or micrometastasis-positive patients develop distant macrometastases, not only bone metastasis but also locoregional relapse and visceral metastasis.^24–26,28,29^ Wiedswang G and colleagues found that 25.0% and 10.2% of bone marrow DTC^+^ breast cancer patients developed bone relapse and liver metastasis versus 7.6% and 4.9% of bone marrow DTC^–^ patients.^30^ The study of Bidard FC and colleagues^26^ also reported that bone marrow DTCs were associated with liver metastasis and locoregional relapse. This clinical evidence supports the possibility that bone marrow DTCs or micrometastases spread into the blood to facilitate secondary dissemination.

#### Adjuvant therapies that eliminate bone marrow tumor cells reduce the risk of extraosseous metastasis

Adjuvant therapies, including bisphosphonate (BP) therapy and chemotherapy, eliminate bone marrow tumor cells, reducing the risk of extraosseous metastasis in patients with bone metastasis or DTCs.

BP therapy is approved as a standard therapy for breast cancer patients with bone metastases since it has been demonstrated to reduce the prevalence of skeletal complications, including bone pain, fractures and hypercalcemia. BP therapy inhibits osteoclastic bone resorption by attaching to hydroxyapatite binding sites on bony surfaces and by decreasing osteoclast progenitor development and recruitment.^31^ Given the effects of BP therapy on blocking the osteoclast-driven “vicious cycle” and growth factor cascade of tumor cells and their potential utility in preventing dormant tumor cell reactivation,^32,33^ BP therapy has been administered to effectively increase DTC clearance and prevent bone metastasis in early-stage breast cancer patients in recent decades.^34^ ASCO-OH (CCO) guidelines recommend starting adjuvant BP therapy early, including intravenous zoledronic acid, oral clodronate and oral ibandronate, for postmenopausal breast cancer patients to prevent cancer recurrence.^35^ In randomized clinical trials, treatment with zoledronic acid for 12 to 24 months resulted in DTC elimination in more than 60% of patients with early-stage breast cancer.^36–39^ Oral ibandronate for 1 year was shown to eliminate bone marrow DTCs in all 17 DTC^+^ breast cancer patients in a pilot study.^40^ Accordingly, breast cancer patients benefit from zoledronic acid, ibandronate or clodronate treatment with significant reductions not only in the incidence of bone metastasis but also in other types of distant metastases and mortality.^27,31,35,41–46^ The Early Breast Cancer Trialists’ Collaborative Group (EBCTCG) reported on a meta-analysis comprising 18 766 patients randomized in trials of adjuvant BPs.^47^ The collaborative meta-analysis suggested that adjuvant BP treatment significantly reduced bone relapse, other distant dissemination and overall mortality in postmenopausal women. This benefit from BPs in postmenopausal women was independent of the type of BP, estrogen receptor (ER) expression, lymph node status, tumor grade, or concomitant chemotherapy.^47^ However, BPs had no significant influence on dissemination to other organs, DFS or OS in DTC^–^ patients.^27^ These clinical data indicate that bone marrow DTC clearance by BP treatment reduces the risk of extraosseous metastasis.

Traditional chemotherapy effectively kills rapidly dividing cancer cells but has limited effects on dormant bone marrow DTCs in a slow-cycling state.^32,33^ Chemotherapeutic agents have been reported to fail to completely clear DTCs in the bone marrow.^27,48^ Adjuvant chemotherapy with docetaxel plus epirubicin or epirubicin plus cyclophosphamide, followed by cyclophosphamide, methotrexate and fluorouracil, has been reported to eliminate 48.3% of CK-positive breast cancer cells in bone marrow and contribute to a decrease in distant metastasis risk and OS.^49^ Patients with remaining bone marrow DTCs after docetaxel treatment had markedly reduced DFS compared with patients with no DTCs after treatment.^50^ Docetaxel-treated patients with no DTCs after treatment had DFS comparable with that of those with no DTCs both before and after chemotherapy.^50^ In addition, chemotherapeutic agents can induce apoptosis of DTCs, which reflects an active response of DTCs to cytotoxic treatment.^27^ Apoptotic DTCs are detected in 48% of patients with primary systemic chemotherapy, leading to less relapse than in patients without apoptosis of DTCs.^27^ Overall, by eliminating cancer cells in bone marrow, BP treatment and chemotherapy contribute to reduced secondary dissemination risk and prolonged survival in breast cancer patients.

### Experimental evidence of bone as a transfer station for the secondary metastasis of breast cancer

In recent years, with the application of several animal models for cancer cell tracing in vivo, the bone microenvironment has been demonstrated to facilitate the seeding of breast cancer cells in bone metastatic lesions to other organs, supporting bone as a booster for the secondary dissemination of cancer cells.

#### Animal experimental methods for tracking the secondary seeding of cancer cells

In animal experiments, bioluminescence imaging (BLI), the evolving CRISPR-barcode system and parabiosis models have been used to track metastasis-to-metastasis seeding of cancer cells and to clarify the time of secondary metastasis.

##### BLI

BLI is the most frequently used method for tracking cancer cells and depicting their distribution in vivo.^51^ Cancer cells, which are modified to express the enzyme luciferase (*luc*), can be detected with an in vivo imaging system in recipient animals when they receive the luciferase substrate. BLI detects photons emitted by an enzymatic reaction in which luciferase catalyzes the production of light from luciferin in the presence of Mg^2+^, ATP and oxygen.^52^ BLI intensity is affected by tissue oxygenation. The BLI signal has been shown to decrease by ∼50% in 0.2% oxygen.^53^ The sensitivity of BLI can also be affected by signal depth.^54^ Light sources closer to the surface of the animal appear brighter than deeper sources because of tissue attenuation properties.^55^ As few as 100 bioluminescent cells can be detected in the peritoneal cavity.^54^ It is estimated that for every centimeter of depth, there is a 10-fold decrease in bioluminescence signal intensity.^56^ Due to the hypoxic state of the bone marrow and the weakening of the bone cortex upon light transmission, a minimum of 1 000 bioluminescent cells are needed for BLI detection.^57^

##### Evolving homing CRISPR barcoding system

An evolving homing CRISPR-barcode system was developed for cellular barcoding and parallel lineage tracing in vivo.^58,59^ It is based on the CRISPR/Cas9 system for engineering evolving DNA barcodes in living cells. This evolving barcoding system uses a homing guide RNA (hgRNA) scaffold to direct the Cas9-hgRNA complex to target the DNA locus of the hgRNA itself.^58^ The homing CRISPR/Cas9 system acts as an expressed genetic barcode that diversifies its sequence. Once Cas9 expression is induced, hgRNA sequences randomly drift, serving as evolving barcodes to generate developmentally barcoded animals in which lineage information is recorded in cell genomes.^16,59^ The diversity of barcodes can be further extracted and rationalized and represented by Shannon entropy, reflecting their lineage histories. The evolving homing CRISPR barcoding system makes it possible to distinguish among independent clones that constitute a primary or metastatic tumor and to learn about clonal heterogeneity during metastasis.^60^

##### Parabiosis models

Parabiosis refers to the condition in which two entire living animals are joined surgically and develop a shared circulatory system.^61^ The surgical technique was first introduced by the French physiologist Paul Bert in the 1860s.^62^ In the beginning, parabiosis surgeries consisted of short skin incisions and suturing together at the flank of each animal. Currently, skin incisions typically extend along the whole body and flank.^62^ Blood circulation interactions can be detected as early as Day 3 after the capillaries of two mice have been connected.^63^ The parabiosis model has been used to reveal crosstalk among resident cells, soluble cytokines and host tumor cells in studies of immune regulation in tumor-bearing animals.^64–67^ In addition, the parabiosis model, mimicking the steps of spontaneous metastases from tumor shedding up to the outgrowth of micrometastases, has been widely employed for metastatic seed tracing of hematogenous metastasis of tumors transplanted from tumor-bearing donor mice to originally tumor-free recipient mice.^16,68–70^

### Experimental evidence of bone as a transfer station for secondary dissemination

Secondary dissemination of breast cancer from an earlier metastasis site to other distant organs has been demonstrated in several experimental models. Bone,^9,16,71–74^ lung,^9,75,76^ axillary lymph node,^9^ skin^9^ and ovary^10^ are potential transfer stations for secondary seeding (Table 1). Bone, as the most frequent first destination of breast cancer metastasis, is considered a robust “launch pad” for secondary metastasis in the metastatic cascade.^77^ An evolutionary analysis and phylogenetic tree based on genomic sequencing of primary breast cancers and metastatic lesions established the probability of linear progression from earlier bone metastasis to subsequent bone metastasis.^9^ In several animal experiments, metastases in the lung and liver have been observed in mice with intratibial injection of breast cancer cells,^71–73^ indicating the possibility of secondary dissemination from bone metastasis. In a xenograft model, a conjugation technology that chemically couples BP to the therapeutic antibody trastuzumab, resulting in the delivery of higher conjugate concentrations to the bone metastatic niche, specifically eliminates bone micrometastases of breast cancer and prevents secondary seeding of multiorgan metastases from bone lesions.^74^

**Table 1:** Experimental evidence of secondary metastasis in breast cancer

From (primary metastasis site)	To (secondary metastasis site)	Experimental evidence	References
Bone	Bone	The probability of secondary bone metastasis from an earlier bone metastasis was established by genomic sequencing and an evolutionary analysis.	^9^
	Lung, liver	Metastases in lung and liver were observed in mice with intratibial injection of breast cancer cells.	^71–73^
	Bone, lung, liver, brain	In a xenograft model, treatment of mice with BP coupled with trastuzumab eliminated bone micrometastases and prevented multiorgan secondary seeding from bone lesions.	^74^
	Bone, lung, liver, kidney, brain	Bone microenvironment was demonstrated to invigorate metastatic seeds for further multiorgan dissemination using a parabiosis model and an evolving barcode system.	^16^
Lung	Liver	The probability of linear progression from an earlier lung metastasis to a subsequent liver metastasis was evaluated by genomic sequencing and an evolutionary analysis.	^9^
	Bone, brain	Cancer cells were observed disseminating to bone marrow and brain from lung metastases generated in mice with tail vein injection of cancer cells.	^75^
	Liver, brain	Secondary seeding from the lungs to liver and brain was presented by bioinformatics analysis based on the SEER (Surveillance, Epidemiology, and End Results) database.	^76^
Axillary lymph node	Bone, brain, liver, skin, colon	The probability of secondary seeding in distant organs from axillary lymph node metastases was established by genomic sequencing and an evolutionary analysis.	^9^
Skin	Bone	The probability of a subsequent bone metastasis from an earlier skin metastasis was established by genomic sequencing and an evolutionary analysis.	^9^
Ovarian	Adrenal gland	Horizontal cross-seeding of an ovarian metastasis to an adrenal gland was observed in a genome-wide phylogenetic analysis.	^10^

Zhang W and colleagues further revealed the impact of the bone microenvironment in facilitating secondary metastasis in mouse breast cancer models.^16^ They demonstrated that bone metastases spread to other organs in experimental models and that the bone microenvironment invigorated metastatic seeds for further multiorgan dissemination.^16^ Bone lesions caused by intrailiac artery injection or intrafemoral injection resulted in multiorgan metastases at late time points in MDA-MB-231 breast cancer models. However, intrailiac vein injection of MDA-MB-231 cells, which delivers more cancer cells directly to the lungs by bypassing the hindlimb, resulted in at least a 10-fold decrease in tumor burden in the lungs and other organs.^16^ In a parabiosis model of bone lesion-carrying donor mice and tumor-free recipient mice, some recipient mice were observed to harbor cancer cells in the lungs, livers, brains, ribs and hindlimbs.^16^ An evolving barcode system was further used to delineate the phylogenetic relationship between initial bone lesions and secondary metastases, revealing spontaneous widespread metastasis-to-metastasis seeding from the bone to visceral organs.^16,77^

Bone metastases have been observed to release a high number of circulating tumor cells (CTCs), probably due to the highly permeable vascular structures or survival advantage in the bone microenvironment.^16^ CTCs exist in the bloodstream as single CTCs or CTC clusters, with the latter featuring a higher capability to facilitate metastatic seeding.^78^ CTCs are intermediary components of the metastatic cascade and are considered to be precursors of metastasis in various cancer types, including breast cancer.^78,79^ Metastasis-derived CTC xenografts in mice have been demonstrated to develop metastases in multiple organs, including bone, brain, liver and lymph nodes.^80^ In addition, CTCs can colonize their tumors of origin in a self-seeding process.^81^ Although self-seeding is a different process from further dissemination from the initial metastatic site, self-seeding can accelerate tumor growth and angiogenesis and increase the migration and invasion capacity, thereby raising the possibility of relapse and secondary metastasis.^81–83^ The phenomenon of tumor self-seeding likely selects for highly aggressive CTCs.^83^ Therefore, bone metastasis-derived CTCs are more efficient than primary tumor-derived CTCs as metastatic seeds in promoting multiorgan dissemination.

### Mechanisms by which the bone microenvironment promotes the secondary metastasis of breast cancer

**Fig. 1:**
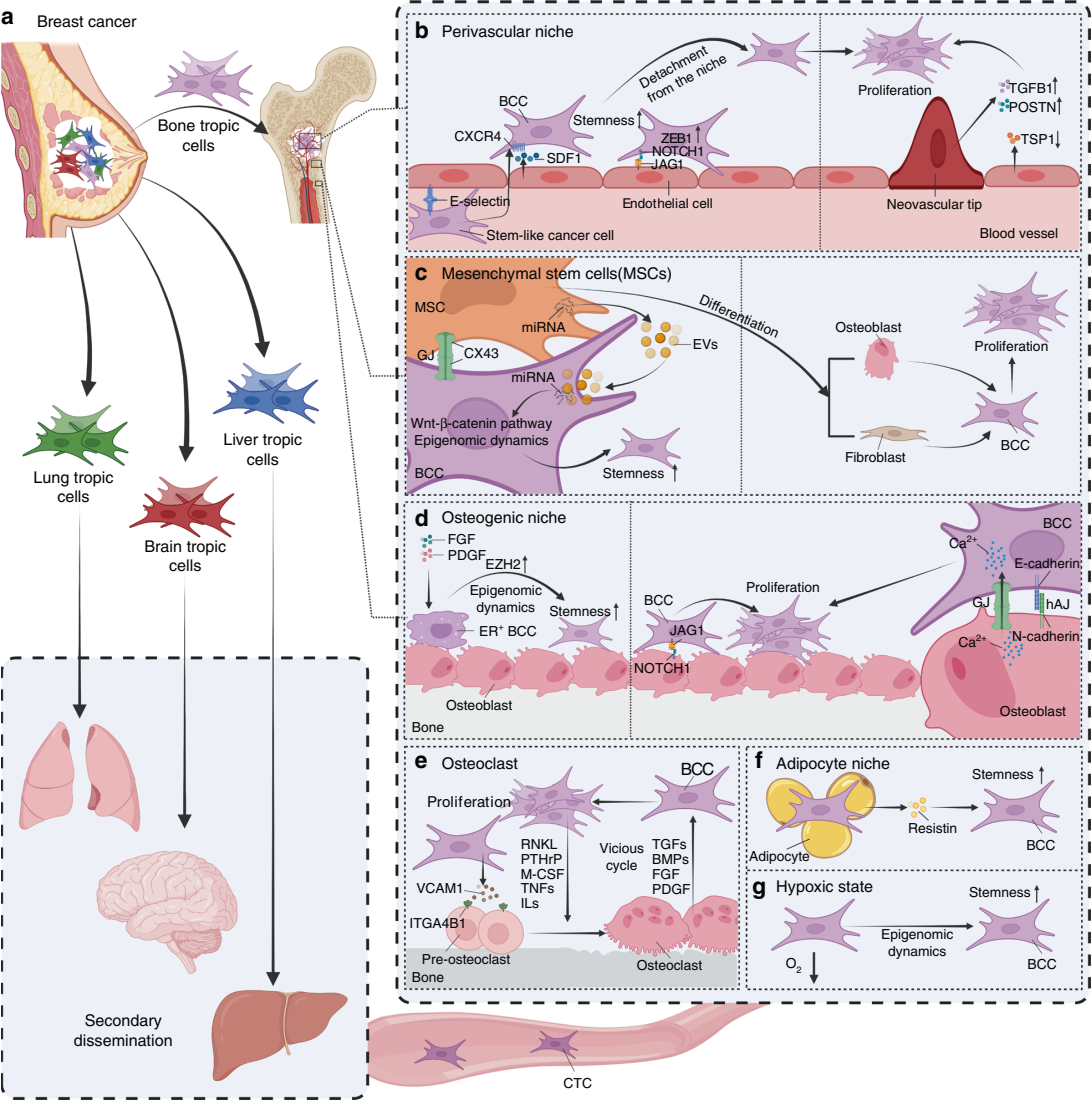
Model of breast cancer cell (BCC) dissemination. **a** Parallel polyclonal model supposing that multiple distant metastases originate from polyclonal organ-specific primary breast cancer cells due to the heterogeneity of cancer cells. **b**–**f** Bone is supposed to be a horizontal cross-metastatic transfer station for secondary dissemination of breast cancer. The perivascular niche (**b**), mesenchymal stem cells (**c**), osteogenic niche (**d**), osteoclasts (**e**), adipocyte niche (**f**) and hypoxic state (**g**) have been considered to regulate the quiescence-proliferation equilibrium by increasing cancer cell stemness, regulating specific signaling pathways and facilitating epigenetic reprogramming. BCC breast cancer cell, JAG1 jagged 1, ZEB1 zinc finger E-box binding homeobox 1, CXCR4 C-X-C chemokine receptor type 4, SDF1 stromal cell-derived factor 1, TGFB transforming growth factor beta, POSTN periostin, TSP1 endothelial-derived thrombospondin 1, MSCs mesenchymal stem cells, EV extracellular vesicle, CX43 connexin 43, FGF fibroblast growth factor, PDGF platelet-derived growth factor, EZH2 enhancer of zeste homolog 2, GJ gap junction, hAJ heterotypic adherens junction, VCAM1 vascular cell adhesion molecule 1, ITGA4B1 integrin α4β1, RANKL nuclear factor-κB ligand, PTHrP parathyroid hormone-related protein, M-CSF macrophage colony stimulating factors, TNF tumor necrosis factors, IL interleukin, BMP bone morphogenetic protein. This figure was created using the BioRender website

Although clonal selection is considered a determinant of organ-specific bone seeding^5,15,71,84^ (Fig. 1a), the bone microenvironment drives secondary multiorgan dissemination in a less organ-specific manner by regulating cancer cell stemness, facilitating epigenetic reprogramming and facilitating immune escape (Fig. 1b–g).

#### The bone microenvironment regulates cancer cell stemness

DTCs in bone marrow enrich the stem cell-like population.^85,86^ The mean proportion of stem-like cells among the DTCs in bone marrow in patients is 72%, while primary tumors consist of less than 10% stem-like cells.^85^ On the one hand, breast cancer stem-like cells demonstrate a high bone-seeking ability.^87^ On the other hand, the bone microenvironment induces and maintains a stem cell-like phenotype in cancer cells.^16,88^ It has been supposed that only stem-like DTCs give rise to clinically detectable metastases.^86^ Accordingly, breast cancer bone metastasis-derived CTCs exhibit a stronger stem cell-like phenotype than CTCs derived from primary breast cancers or lung metastases in mice.^16,77^ The majority of CTCs die during the process of dissemination as a result of biological and physical constraints such as shear stress and immune surveillance.^89^ Only CTCs harboring a stem cell-like phenotype and tumor initiation capacity are thought to possess the clonogenic potential to seed new metastatic lesions.^90,91^ This evidence demonstrates that bone metastasis-derived CTCs, due to their stem-like properties, act as efficient seeds in secondary dissemination.

Cancer cells with stem cell-like properties are often more quiescent than other cancer cells but are persistent and therapeutically resistant.^77,92^ Specific niches in the bone microenvironment have been considered regulators of quiescence-proliferation equilibrium (Table 2). Dormant and proliferating breast cancer cells in the bone microenvironment occupy distinct areas and niches.^88,93^ Tumor cells may shuttle between specific niches and interact with different partners in the bone microenvironment, leading to phenotypic transitions among quiescent, proliferative and secondary dissemination-promoting states.

**Table 2:** Crosstalk and molecular interactions between the bone microenvironment and breast cancer cells

Crosstalk	Molecular interaction	Effects on the bone microenvironment	Effects on tumor cells	References
Perivascular niche/tumor cells	E-selectin/E-selectin	/	Stem-like cancer cell homing to the bone marrow	^93^
	SDF1/CXCR4	/	Being anchored to the perivascular niche and being prevented from entering into circulation	^93^
	JAG1/NOTCH	/	Stemness acquisition by activating the NOTCH1-ZEB1 pathway	^94^
	Endothelium-derived TSP1	/	Quiescence	^95^
	Endothelium-derived TGFB1 and POSTN	/	Proliferation	^95^
MSCs/tumor cells	CX43-dependent gap junction	/	Dormancy and evasion from immune surveillance	^99^
	MSC-derived extracellular vesicles loaded with miRNAs	/	Dormancy in the Wnt-catenin-dependent pathway	^96,98^
Osteoblasts/tumor cells	N-cadherin/E-cadherin heterotype adherens junction	/	Proliferation by activating the mTOR pathway	^102^
	CX43-dependent gap junction (Ca^2+^ flow)	/	Proliferation	^104^
	NOTCH/JAG1	IL-6 release from osteoblasts leading to osteoclast activation	Proliferation by activating the NOTCH pathway	^107,108^
	FGF/FGFR PDGF/PDGFR	/	Stemness increase and endocrine resistance mediated by EZH2-mediated epigenomic reprogramming	^88^
Bone matrix/tumor cells	Bone matrix protein/integrin α5	/	Proliferation	^105,106^
Osteoclasts/tumor cells	Integrin α4β1/VCAM-1	Osteoclast progenitor recruitment and activation	Activating indolent tumor cells	^112^
	Vicious cycle	Osteoclast activation, bone resorption and growth factor release (TGFB, BMPs, FGF and PDGF)	Proliferation and secretion of osteoclastogenic factors (RANKL, PTHrP, M-CFS, TNFs and ILs)	^113–115^
Adipocytes/tumor cells	Resistin/TLR4	/	Stemness acquisition through activation of NF-κB and STAT3	^118^
HSCs and HPCs/tumor cells	Tumor-derived cytokines (GM-CSF, G-CSF, IL6 and IFNs)	HSC and HPC differentiation into MDSCs	Immune escape due to the suppression of T-lymphocyte and NK-cell responses	^130–135^
Erythroid precursor cells/tumor cells	Tumor-derived GM-CSF	Erythroid-to-myeloid transdifferentiation	Immune escape from T-cell-mediated antitumor responses	^129^
Macrophages/tumor cells	IL6 and WISP1 secreted from JAG1-expressing cancer cells	Macrophage recruitment, activation, CD14 and CD93 secretion	Immune escape from the killing of CD8^ +^ T lymphocytes	^137^
NK cells/latency competent cancer cells	ULBPs/NK activating receptor	Reduced tumor cell recognition by NK cells	Evasion of NK-cell-mediated clearance	^126^

##### Perivascular niche

Dormant cancer cells are predominantly found in sinusoidal perivascular niches; however, proliferating cancer cell clusters have been identified in physically distinct, lateral, nonsinusoidal regions of the bone marrow.^93^ Vascular E-selectin has been identified as a mediator of CD44^+^CD24^−/low^ stem-like cancer cell homing to the bone marrow; furthermore, stromal cell-derived factor 1 (SDF1) anchors cancer cells to the perivascular niche by interacting with C-X-C chemokine receptor type 4 (CXCR4).^93^ The perivascular niche maintains the stemness of breast cancer cells by upregulating zinc finger E-box binding homeobox 1 (Zeb1) expression mediated by the interaction of endothelial Jagged 1 (JAG1) with cancer cell-derived NOTCH1.^94^ However, neovascular tips, which are characterized by reduced endothelial-derived thrombospondin 1 (TSP1) expression and enhanced expression of the protumor factors transforming growth factor beta 1 (TGFB1) and periostin (POSTN), lose the tumor-suppressive nature of the perivascular niche and accelerate breast cancer cell proliferation.^95^ Overall, the stable perivascular niche maintains cancer cell quiescence, whereas the sprouting neovasculature induces dormant cancer cell reactivation and micrometastatic outgrowth^95^ (Fig. 1b).

##### Mesenchymal stem cells (MSCs)

MSCs, which comprise the first set of bone microenvironment niche cells encountered by breast cancer cells, change the behavior of cancer cells^96^ (Fig. 1c). MSCs induce breast cancer cell dedifferentiation into stem-like cells, support cancer cell survival and instruct cancer cells into dormancy in the bone marrow.^96–98^ In an in vitro 3D coculture model that mimics the cellular interactions of MSCs and cancer cells, cancer cells under duress are observed to obtain a stem cell-like phenotype and enter dormancy after cannibalizing MSCs.^97^ MSCs can also communicate with breast cancer cells through connexin 43 (CX43)-dependent gap junctions (GJs), therefore supporting the survival of quiescent cancer cells.^99^ Furthermore, MSC-secreted extracellular vesicles (EVs) have been shown to instruct breast cancer cells into dormancy by inducing breast cancer cell dedifferentiation into a stem cell-like population in a Wnt-catenin-dependent pathway.^96,98^ In addition, MSCs are multipotent cells capable of differentiating into fibroblasts, osteoblasts, adipocytes or chondrocytes. MSC-differentiated cancer-associated fibroblasts support the survival and growth of cancer cells.^100,101^ MSC-differentiated osteogenic cells might also play roles in supporting the survival and colonization of breast cancer cells in the bone marrow.

##### Osteogenic niche

The osteogenic niche is a microenvironment exhibiting active osteogenesis in which osteogenic cells, bone matrix and tumor cells crosstalk with each other.^102,103^ It has been supposed that the osteogenic niche promotes the transition of breast cancer cells from an indolent phenotype to an aggressive phenotype^16,102,104^ (Fig. 1d). In the osteogenic niche, the bone matrix increases cancer cell proliferation in an integrin-dependent manner^105,106^; osteoblasts and mature osteocytes transfer cancer cell growth advantages via direct connections or release of cytokines.^107–109^ Heterotypic adherens junctions (hAJs) involving cancer-derived E-cadherin and osteogenic N-cadherin^102^ and GJs transferring Ca^2+^ flow from osteogenic cells to cancer cells^104^ drive the formation of micrometastases in bone from DTCs. JAG1-expressing cancer cells can also obtain a growth advantage in the bone microenvironment via JAG1-NOTCH-dependent crosstalk with the osteogenic niche.^107,108^ Bado IL and colleagues have demonstrated that the osteogenic niche enhances the phenotypic plasticity and stemness of metastatic estrogen receptor-positive (ER^+^) breast cancer cells through enhancer of zeste homolog 2 (EZH2)-mediated epigenomic reprogramming.^88^ The osteogenic niche transiently and reversibly reduces ER expression and leads to stem-like properties and endocrine resistance in bone micrometastasis margins next to the bone matrix. However, ER downregulation and endocrine resistance are partially attenuated in cancer cells away from the osteogenic niche.^88^ These transient and reversible phenotypic changes in ER^+^ cancer cells in the bone microenvironment may lead to the transition from bone colonization to aggressive secondary metastatic spread. Fibroblast growth factor receptor (FGFR) and platelet-derived growth factor receptor (PDGFR) signaling pathways in the osteogenic niche have further been discovered to contribute to phenotypic changes by increasing EZH2 expression.^88^ EZH2, which is essential in stem cell self-renewal, has been linked to stem cell-like properties and breast cancer progression.^110,111^ Moreover, EZH2 in cancer cells orchestrates the effects of the bone microenvironment on secondary metastasis.^16^ Transient treatment with an EZH2 inhibitor or inducible knockdown of EZH2 in cancer cells dramatically decreases secondary metastasis from bone metastatic lesions.^16^

##### Osteoclasts

Breast cancer cells recruit osteoclast progenitors and elevate osteoclast activity by interacting with vascular cell adhesion molecule 1 (VCAM1) and the cognate receptor integrin α4β1.^112^ Activated osteoclasts further activate indolent tumor cells by driving a “vicious cycle” (Fig. 1e). Tumor cells in the bone secrete soluble osteoclastogenic factors, including nuclear factor-κB ligand (RANKL), parathyroid hormone-related protein (PTHrP), macrophage-colony stimulating factor (M-CSF), tumor necrosis factors (TNFs) and several interleukins (ILs), resulting in osteoclast activation and an increase in osteoclastic bone resorption. This process disrupts bone homeostasis and induces the release of growth factors, including TGF-β, bone morphogenetic proteins (BMPs), FGF and PDGF, from the degraded bone matrices, contributing to tumor growth and progression in the bone. This feedback loop increases the proliferation and aggressive phenotype of tumor cells.^113–115^

In addition, the adipocyte niche (Fig. 1f) and the hypoxic state (Fig. 1g) in the bone marrow serve as promoters of stem-like properties and dormancy of breast cancer cells.^116–118^

#### Epigenetic reprogramming in cancer cells interacting with the bone microenvironment contributes to secondary dissemination

Due to the phenotypic plasticity and reversible stemness of cancer cells in the bone microenvironment, transient and reversible epigenetic reprogramming in cancer cells is considered as a contributor to secondary dissemination from bone lesions. Breast cancer cells in bone metastases are enriched with trimethylation on H3 lysine (K) 27 (H3K27me3), and H3K27me3 is reversible after several passages in vitro, suggesting the impact of the bone microenvironment.^16,88^ Epigenomic reprogramming beyond H3K27me3 is triggered by EZH2.^119^ EZH2 is a histone methyltransferase of polycomb repressive complex 2 (PRC2) catalyzing the methylation of H3K27 in target gene promoters.^119,120^ The role of H3K27me3 in secondary dissemination is in accordance with the increasing EZH2 expression and the roles of EZH2 in the maintenance of stem-like properties and secondary dissemination of cancer cells in the bone microenvironment. Sandiford OA and colleagues investigated a ten-eleven translocation (TET)-mediated DNA demethylation process and stem cell-like property acquisition in cancer cells treated with MSC-secreted EVs.^96^ Moreover, the hypoxic state of the bone marrow can also lead to epigenetic dynamics in cancer cells,^116,121,122^ although hypoxia-induced epigenetic changes have not been shown to be associated with secondary dissemination. Hypoxia-driven epigenetic dynamics can promote cancer cell stem-like properties and support cancer cell dormancy by hypermethylation of promoter regions associated with tumor suppressor genes and downregulation of TET activity.^116,123^

#### Bone serves as a shelter for cancer cells escaping immune surveillance

**Fig. 2:**
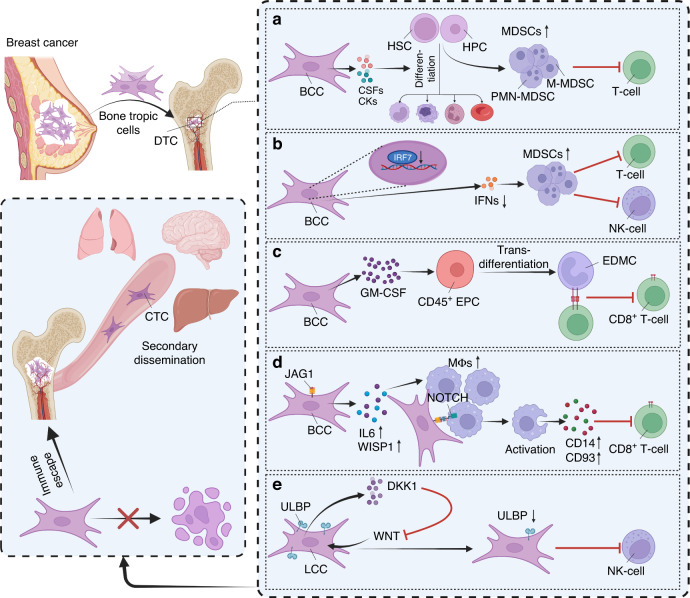
The bone marrow microenvironment facilitates disseminated tumor cell (DTC) escape from immune surveillance. **a** Breast cancer cell (BCC)-derived colony-stimulating factors (CSFs) and cytokines (CKs) induce the rapid generation of myeloid-derived suppressor cells (MDSCs) from hematopoietic stem cells (HSCs) and hematopoietic progenitor cells (HPCs) in bone marrow. Monocytic MDSCs (M-MDSCs) and polymorphonuclear MDSCs (PMN-MDSCs) suppress T lymphocyte (T cell) activity. **b** Silencing of the interferon regulatory factor 7 (IRF7) pathway in breast cancer cells, reducing interferons (IFNs), restricts immunosurveillance by selective modulation of MDSCs and natural killer (NK) effectors in the bone marrow. **c** Tumor-derived granulocyte-macrophage colony-stimulating factor (GM-CSF)-induced erythroid precursor (EPC)-differentiated myeloid cells (EDMCs) mediate immunosuppression for cancer cells to escape surveillance. **d** Jagged 1 (JAG1)-overexpressing BCCs increase the secretion of interleukin-6 (IL6) and WNT1-inducible signaling pathway protein 1 (WISP1), helping to recruit macrophages (Mϕs). Recruited Mϕs are activated in the NOTCH pathway and increase the secretion of CD14 and CD93 to inhibit CD8^+^ T-cell activation and decrease the cytotoxic killing of tumor cells. **e** Latency competent cancer (LCC) cells, including disseminated tumor cells (DTCs) in bone marrow, escape NK cell-mediated clearance by inhibition of the WNT pathway and downregulation of UL16-binding protein (ULBP) ligands. This figure was created using the BioRender website

The bone marrow has been identified as an immunosuppressive microenvironment for bone colonization in a variety of tumors, characterized by the accumulation of myeloid-derived suppressor cells (MDSCs), exhausted cytotoxic T cells, abundant regulatory T cells (Tregs), and immune checkpoint inhibitor treatment resistance, and is orchestrated by the overexpression of immunosuppressive cytokines.^124–129^ The bone marrow microenvironment is where hematopoietic stem cells (HSCs) and hematopoietic progenitor cells (HPCs) exist. HSCs in the bone marrow give rise to two main types of cells: myeloid lineage cells and lymphoid lineage cells. Immune cells originate from HSCs in the bone marrow and differentiate into mature cells in specific immune organs. In the bone marrow, tumor cells crosstalk with HSCs and HPCs to form a potent immunosuppressive microenvironment to facilitate tumor cell escape from immune surveillance (Fig. 2a).

Tumor-derived factors (colony-stimulating factors, proinflammatory cytokines, and others) can regulate the differentiation of HSCs and HPCs in the bone marrow and subsequently contribute to the accumulation of MDSCs.^130–133^ MDSCs are defined as a group of immature CD11b^+^GR1^+^ cells that inhibit tumor-specific immune responses, which include precursors of macrophages, granulocytes, dendritic cells (DCs) and myeloid cells.^134^ Two main MDSC populations have been characterized: monocytic MDSCs (M-MDSCs) and polymorphonuclear MDSCs (PMN-MDSCs). M-MDSCs correlate with the suppression of T-lymphocyte activation, and PMN-MDSCs suppress CD8^+^ T cells predominantly by producing reactive oxygen species (ROS)^134^ (Fig. 2a, Table 2). The percentage of MDSCs in the bone marrow has been found to be far greater than that in the lungs and mammary glands.^135^ An innate immune pathway of interferon regulatory factor 7 (IRF7) silencing has been identified to facilitate the immune escape of breast cancer cells and promote bone metastasis via selective modulation of MDSCs and natural killer (NK) effectors in the bone marrow^135^ (Fig. 2b).

Tumor-induced erythroid-to-myeloid transdifferentiation has also been demonstrated to be an immunosuppressive mechanism to escape surveillance and curtail anti-PD-1/PD-L1 treatment efficacy.^129^ CD45^+^CD71^+^TER119^+^ erythroid precursor cells have been shown to exert immunosuppressive effects on CD8^+^ T cells.^136^ The same research group further demonstrated that tumors induce erythroid precursor cells to differentiate into tumor-associated myeloid cells.^129^ Erythroid differentiated myeloid cells (EDMCs; CD45^+^CD235a^+^CD71^+^CD11b^+^CD33^+^HLA-DR^─^ in cancer patients and CD45^+^Ter119^+^CD71^+^CD11b^+^Gr1^+^ in tumor-bearing mice) correlate with an immunosuppressive tumor microenvironment with an attenuated T-cell-mediated antitumor response^129^ (Fig. 2c, Table 2).

Meng J and colleagues determined tumor-derived JAG1-mediated cancer cell immune evasion progression.^137^ JAG1-overexpressing breast cancer cells increase the expression and secretion of multiple cytokines, including IL6 and WNT1-inducible signaling pathway protein 1 (WISP1), to help recruit macrophages into the tumor microenvironment. Recruited macrophages are activated in the NOTCH pathway and increase the secretion of CD14 and CD93 to inhibit CD8^+^ T-cell proliferation, increase PD-1^+^CD8^+^ cytotoxic T-cell exhaustion and decrease cytotoxic killing of tumor cells (Fig. 2d, Table 2). The authors supposed that JAG1-expressing breast cancer cells might be selected based on their survival of early adaptive immune surveillance, and then, these cells may colonize the bone by interacting with bone host cells.^107,108^ However, another reasonable explanation is that JAG1-expressing tumor cells might be selected and obtain a proliferation advantage in the bone microenvironment and then escape immune surveillance, leading not only to bone colonization but also to secondary dissemination.

In addition, latency competent cancer (LCC) cells, including DTCs in the bone marrow, show stem cell-like characteristics and remain quiescent.^126^ LCC cells isolated from breast cancer cells self-impose a slow-cycling state with broad downregulation of UL16-binding protein (ULBP) ligands for NK cells and evasion of NK-cell-mediated clearance through autocrine inhibition of the WNT pathway^126^ (Fig. 2e, Table 2). Overall, once tumor cells spread to the bone marrow, tumor cells, either DTCs or bone metastasis cells, promote the formation of an immunosuppressive microenvironment, facilitating their evasion of immune surveillance and promoting secondary dissemination.

## Conclusions and future perspectives

In conclusion, remarkable progress in the knowledge regarding bone as a transfer station for the secondary dissemination of breast cancer cells has been made in the past decade due to the application of high-throughput sequencing-based evolutionary analyses and the improvement of experimental methods for tracking cancer cell seeding in animal models. However, much work is still needed to clarify the role of the bone microenvironment in the secondary dissemination of breast cancer and to develop effective blocking strategies.

### Do DTCs in bone marrow spread to other organs prior to bone colonization?

Breast cancer cell dissemination to bone is an early event in tumor progression. Bone DTCs can remain asymptomatic for years before progressing to osteolytic overt lesions. It seems possible that cancer cells, in the long-term interaction with the bone microenvironment, might acquire the capability to spread to other organs prior to symptomatic bone metastasis. Moreover, luminal-like cancer has a high risk of bone colonization; however, basal-like cancer is prone to visceral metastasis, although the prevalence of bone marrow DTCs is similar in patients with luminal-like cancer and patients with basal-like cancer. This clinical feature raises another question: do bone marrow DTCs of basal-like cancer spread to other organs rather than colonize the bone? The answer will determine whether quiescent DTCs in the bone marrow should be targeted for treatment. Furthermore, the potential mechanism by which the bone microenvironment alters the bone colonization ability of bone DTCs should be considered.

### Is secondary dissemination determined by the loss of bone-specific metastatic gene expression?

Organ-specific metastatic gene expression signatures have been identified as drivers of the parallel polyclonal seeding of breast cancer cells.^5,138,139^ Bone-specific metastatic genes have significantly enhanced our understanding of the bone tropism of breast cancer “seeds”.^6,71,84^ However, secondary dissemination from bone, which implies a reduction in the bone-specific metastatic phenotype, might indicate the possibility of decreasing bone metastasis-specific gene expression levels. These changes in gene expression levels may be the result of epigenetic dynamics in cancer cells interacting with the bone microenvironment. Furthermore, because bone-only metastasis is less lethal than visceral metastasis, whether cancer cells can be limited in bone by blocking the loss of bone-specific metastatic gene expression remains to be determined. This will need to be demonstrated in future research.

### Prevention of secondary dissemination

Bone marrow DTCs can survive in a dormant state for several years. It is unclear which factors disturb the balance in the bone microenvironment to facilitate secondary dissemination. The factors that limit cancer cells in the bone to developing bone-only colonization are also unclear. The answers will reveal potential prognostic factors for breast cancer patients and be helpful in developing strategies for preventing metastasis.

Current bone-targeting therapies aim to inhibit bone resorption, reduce complications, and prolong survival. Research on secondary dissemination from bone raises the need for treatments targeting dormant DTCs and asymptomatic micrometastases. Bone DTCs and micrometastases in a slow-cycling state usually present resistance to traditional chemotherapy, which effectively kills rapidly dividing cells.^32,33^ The perivascular niche in the bone marrow microenvironment also protects DTCs from chemotherapy, independent of cell cycle status.^48^ Although adjuvant BP therapy has been shown to be effective in eliminating bone marrow DTCs and is recommended for use in preventing breast cancer recurrence according to ASCO-OH (CCO) guidelines,^35^ new therapeutic strategies should be developed in the future to achieve therapeutic sensitivity of quiescent DTCs without inducing DTC proliferation or increasing cytotoxicity. In the future, therapeutic strategies preventing secondary dissemination will focus on targeting the molecular interaction of tumor cells with the bone microenvironment. Several research groups have attempted to prevent bone metastasis and secondary metastasis by blocking the molecular crosstalk between DTCs and the bone microenvironment. Disruption of the JAG1-NOTCH interaction and gap junction inhibition, targeting the connection between cancer cells and osteoblasts, reduce bone metastasis.^104,107,108^ Antibodies against vascular cell VCAM1 and integrin α4 disrupt the interaction between cancer cells and osteoclast progenitors and effectively inhibit bone metastasis progression.^112^ Inhibiting the integrin-mediated interaction between DTCs and the perivascular niche sensitizes DTCs to chemotherapy without inducing DTC proliferation.^48,140^ EZH2 inhibitors abolish the stemness of cancer cells conferred by the bone microenvironment and block secondary metastatic spread.^16^ Due to these research advances, with the understanding of crosstalk between the bone microenvironment and cancer cells, therapeutic strategies targeting crosstalk will be developed and used for individualized clinical therapy.
